# Caution Should be Used in the Recognition of Adult-Onset Autoinflammatory Disorders: Facts or Fiction?

**DOI:** 10.3389/fimmu.2013.00096

**Published:** 2013-04-23

**Authors:** Luca Cantarini, Orso Maria Lucherini, Donato Rigante

**Affiliations:** ^1^Research Center of Systemic Autoimmune and Autoinflammatory Diseases, Unit of Rheumatology, Policlinico Le Scotte, University of SienaSiena, Italy; ^2^Institute of Pediatrics, Università Cattolica Sacro CuoreRome, Italy

Autoinflammatory disorders (AIDs) are a group of inherited diseases of innate immunity, characterized by seemingly unprovoked inflammation recurring with variable rhythmicity and involving skin, serosal membranes, synovial membranes, and gastrointestinal tube, with reactive amyloidosis as a potential severe long-term complication (Touitou and Koné-Paut, 2008). They can be categorized in hereditary monogenic disorders and multi-factorial polygenic disorders, encompassing an expanding number of conditions, as the well-known periodic fever, aphthosis, pharyngitis, and adenitis (PFAPA) syndrome (Masters et al., 2009).

Monogenic AIDs are caused by mutations in genes encoding proteins involved in the regulation or activation of the inflammatory response, and include familial Mediterranean fever (FMF), tumor necrosis factor receptor-associated periodic syndrome (TRAPS), mevalonate kinase deficiency syndrome, the family of cryopyrin-associated periodic syndromes (CAPS), which in turn include familial cold autoinflammatory syndrome (FCAS), Muckle–Wells syndrome and neonatal onset multisystem inflammatory disease, NLRP12-associated autoinflammatory disorder (NLRP12AD), PAPA (pyogenic arthritis, pyoderma gangrenosum, acne) syndrome, Majeed's syndrome, deficiency of interleukin-1 receptor antagonist, and – lastly – Blau's syndrome. Recurrent multi-district inflammatory flares, which typically alternate with symptom-free intervals characterized by complete well-being and normalization of acute phase reactants, are the most striking marker of AIDs. The vast majority of these conditions are related to the activation of the interleukin-1 pathway, which results in a unifying common pathogenetic mechanism (Rigante, 2012). Table 1 summarizes their genetic characteristics, inheritance patterns, and the most peculiar clinical manifestations. The recognition of AIDs derives from the combination of clinical data, evaluation of acute phase reactants, clinical efficacy in response to specific drugs, and identification of specific mutations in the causative genes (Federici et al., 2006; Gattorno et al., 2008; Cantarini et al., 2010a, 2011, 2012a,b; Muscari et al., 2012). Genetic testing remains a feasible tool to corroborate the clinical diagnosis of AIDs.

**Table 1:** **Summary of the main genetic and clinical features of monogenic autoinflammatory disorders**.

Disease	*Gene* locus	Protein	Inheritance	Prominent clinical features
				
FMF	*MEFV* 16p13.3	Pyrin	AR	Fever, serositis, arthralgias or arthritides, erysipelas-like eruption on the legs, amyloidosis in untreated or resistant or non-compliant patients
TRAPS	*TNFRSF1A* 12p13	Tumor necrosis factor receptor 1	AD	Fever, migrating muscle and joint involvement, arthralgias or arthritides, serosal involvement, steroid responsiveness of febrile attacks, conjunctivitis, periorbital edema, amyloidosis
MKD	*MVK* 12q24	Mevalonate kinase	AR	Fever, polymorphous rash, arthralgias, abdominal pain, diarrhea, lymphnode enlargement, headache, splenomegaly, oral aphthosis
FCAS				Fever, cold-induced urticaria-like rash, conjunctivitis, arthralgias
MWS	*NLRP3* 1q44	Cryopyrin	AD	Fever, urticaria-like rash, conjunctivitis, arthralgias, neurosensorial deafness, amyloidosis
NOMID				Fever, urticaria-like rash, uveitis, papilledema, deforming arthritides involving large joints (knees), aseptic chronic meningopathy, neurosensorial deafness, amyloidosis
NLRP12AD	*NLRP12* 19q13	Monarch-1	AD	Fever, arthralgias, cold-induced urticaria-like rash
BS	*NOD2* (*CARD15*) 16q12.1–13	NOD2	AD	Granulomatous dermatitis with ichthyosis-like changes, granulomatous polyarthritis, camptodactyly, recurrent panuveitis, intermittent fevers
PAPAs	*PSTPIP1* 15q24–q25.1	CD_2_ antigen-binding protein 1	AD	Pyoderma gangrenosum, cystic acne, sterile pyogenic oligoarthritis
MS	*LPIN2* 18p11.31	Lipin 2	AR	Recurrent multifocal osteomyelitis, dyserythropoietic anemia, neutrophilic chronic dermatosis
DIRA	*IL1RN* 2q14	Interleukin-1 receptor antagonist	AR	Multifocal osteomyelitis, diffuse pustular rash with neonatal onset

*FMF, familial Mediterranean fever; TRAPS, tumor necrosis factor receptor-associated periodic syndrome; MKD, mevalonate kinase deficiency syndrome; FCAS, familial cold autoinflammatory syndrome; MWS, Muckle–Wells syndrome; NOMID, neonatal onset multisystem inflammatory disease; NLRP12AD, NLRP12-associated autoinflammatory disorder; BS, Blau syndrome; PAPAs, pyogenic arthritis, pyoderma gangrenosum, acne syndrome; MS, Majeed syndrome; DIRA, deficiency of interleukin-1 receptor antagonist; AR, autosomal recessive; AD, autosomal dominant*.

Most of these disorders are manifest in childhood, but a certain number of patients may experience disease onset during adulthood. To date, among monogenic AIDs a late disease onset has been described in FMF (Sohar et al., 1967; Sayarlioglu et al., 2005; Cantarini et al., 2010b), TRAPS (Aksentijevich et al., 2001; Dodé et al., 2002; Aganna et al., 2003; Cantarini et al., 2012c), CAPS (Vitale et al., 2012), and NLRP12AD (Borghini et al., 2011).

Adult-onset FMF is fairly rare, however it has been reported as late as age 65 (Cantarini et al., 2010b), and advanced age is not an exclusion criterion (Livneh et al., 1997): this picture is mainly related to low-penetrance mutations, giving rise to a milder disease, generally similar to that of younger patients (Sayarlioglu et al., 2005).

On the contrary, a more significant variability in terms of clinical phenotype may be observed in TRAPS (Aksentijevich et al., 2001; Dodé et al., 2002; Aganna et al., 2003; Ravet et al., 2006; Cantarini et al., 2009, 2010c,d,e,f,g; Rigante et al., 2011; Brizi et al., 2012): this heterogeneity is largely related to the wide spectrum of known *TNFRSF1A* mutations, which can be distinguished into high-penetrance and low-penetrance variants (Touitou et al., 2004). As in FMF, adult-onset of symptoms is usually related to low-penetrance mutations, which are associated with feeble clinical signs and a lower risk of amyloidosis (Aksentijevich et al., 2001; Dodé et al., 2002; Aganna et al., 2003). In addition, TRAPS patients carrying low-penetrance *TNFRSF1A* variants may show atypical clinical manifestations and symptoms that mimic other AIDs and/or autoimmune diseases, such as idiopathic recurrent acute pericarditis, thus hindering differential diagnosis (Cantarini et al., 2009, 2010d,e,g, 2012a; Rigante et al., 2011). These alleles have also been described in patients with recurrent inflammatory attacks who lack the most typical TRAPS manifestations, even when the duration of fever episodes is short and might resemble FMF, and even in healthy controls (Dodé et al., 2002; Ravet et al., 2006; Cantarini et al., 2010a, 2011; Muscari et al., 2012). Their pathogenetic role and the genotype-phenotype correlation are still a huge matter of debate. Moreover, the absence of segregation in some families suggests the existence of other genes with a non-permissive power on AIDs expression (D'Osualdo et al., 2006).

On the other hand, CAPS are primarily characterized by the onset of symptoms during early-infancy: nevertheless, we have recently described a case series of patients presenting FCAS-like symptoms, carrying the low-penetrance Q703K mutation in the *NLRP3* gene, all characterized by disease onset during adulthood, and with clinical manifestations triggered or worsened by cold exposure (Vitale et al., 2012). Other *NLRP3* mutations of unknown pathogenetic significance can also be found both in patients with recurrent inflammatory attacks and in healthy controls: thus, their causal role remains doubtful and the question of whether these variants are low-penetrance disease-associated mutations or asymptomatic polymorphisms has been often raised (Aróstegui et al., 2004; Aksentijevich et al., 2007; Verma et al., 2008). As in TRAPS, it has been suggested that these alleles might exert a possible proinflammatory effect, causing an inflammatory phenotype in concomitance with other eventual environmental and/or genetic factors.

Also NLRP12AD has been shown to be characterized by neonatal or early-infancy onset (Goldbach-Mansky, 2012). However, Borghini et al. (2011) have recently described a 32-year-old woman found to be a carrier of the D294E *NLRP12* mutation: this patient experienced FCAS-like symptoms since the age of 20. In agreement with these findings, we recently diagnosed a 27-year-old Caucasian woman with NLRP12AD and this patient carried the F402L mutation presenting a daily low-grade fever (<38°C) since the age 22 (*unpublished data*). Hence, we underscore that patients carrying *NLRP12* mutations might undoubtedly display a disease onset during adulthood.

The differential diagnosis of AIDs can be complicated by PFAPA syndrome, which frequently occurs in pediatric patients. This syndrome does not have a documented genetic basis, and spontaneous resolution of fever episodes is commonly observed a few years after symptom onset (Marshall et al., 1987, 1989; Thomas et al., 1999). Recent medical literature has included dozens of suspected cases in adults as well, suggesting that it should be taken into consideration also in adults (Cavuoto and Bonagura, 2008; Padeh et al., 2008; Colotto et al., 2011; Cantarini et al., 2012d,e; Cazzato et al., 2013).

In conclusion, although little data is available in the literature in comparison with the medical amount of clinical notes related to the pediatric population, the increasingly frequent reports of adult patients with AIDs is gradually allowing a more extensive and detailed information regarding their potential belated onset, genotype-phenotype correlations, overall prognosis, and management of therapy. Both a delayed diagnosis during adulthood and adult-onset of symptoms are more and more observed: in these cases the presence of low-penetrance mutations, giving nuanced clinical pictures in comparison with children, can be advocated. Low-penetrance mutations may also be responsible for oligosymptomatic diseases in some cases, and for the appearance of atypical clinical manifestations in others, but may even function as susceptibility alleles to inflammation, rather than disease-associated mutations (Dodé et al., 2002; Ravet et al., 2006; Cantarini et al., 2010d, 2012a). Indeed, we suggest caution in the interpretation of low-penetrance mutations in probands with suspected AIDs in order to avoid false positive diagnoses and overtreatment, given the high frequency of healthy carriers and the influence of additional, still unknown, genetic and/or environmental modifying factors.
